# Lymphocyte dynamics as the central mediator in osimertinib-induced CD4^+^ T-cell depletion, fulminant cytomegalovirus pneumonitis, and progressive pulmonary fibrosis: a case report

**DOI:** 10.3389/fimmu.2025.1702074

**Published:** 2025-12-17

**Authors:** Liya Zhu, Dan Liu, Jie Peng, Jinzhi Lu

**Affiliations:** 1 Department of Infectious Diseases, The First Affiliated Hospital of Yangtze University, Jingzhou, Hubei, China; 2 Department of Obstetrics and Gynecology, The First Affiliated Hospital of Yangtze University, Jingzhou, Hubei, China; 3 Department of Radiology, The First Affiliated Hospital of Yangtze University, Jingzhou, Hubei, China; 4 Department of Laboratory Medicine, The First Affiliated Hospital of Yangtze University, Jingzhou, Hubei, China

**Keywords:** CD4+ T-cell, CMV pneumonia, diagnostic error, drug-induced lung injury, lymphocytopenia, osimertinib, pulmonary fibrosis

## Abstract

Osimertinib-induced severe lymphocytopenia can create a profound immunodeficiency state, facilitating opportunistic infections and progressive fibrotic lung disease. A 75-year-old female with EGFR-mutant NSCLC developed respiratory failure with diffuse ground-glass opacities and profound lymphocytopenia (ALC 0.48×10^9^/L). Overreliance on BAL-NGS detection of Mycobacterium avium complex delayed diagnosis of cytomegalovirus pneumonia. Guideline-discordant erlotinib rechallenge accelerated lymphocyte depletion, culminating in high-grade CMV viremia with CD4^+^ lymphocytopenia (0.16×10^9^/L) and irreversible pulmonary fibrosis despite ganciclovir-induced virologic clearance. This case demonstrates an immune-fibrotic axis wherein TKI-induced lymphocytopenia enables CMV pneumonitis and fibrotic remodeling. Lymphocytopenia in this setting mandates urgent viral exclusion before attributing injury to drug toxicity and precludes TKI rechallenge during active infection or severe immunosuppression. BAL-NGS requires rigorous clinicoradiologic correlation.

## 1 Introduction

Osimertinib, a first-line therapy for EGFR-mutant non-small cell lung cancer (NSCLC), induces severe lymphocytopenia (Grade ≥3: 6.1%) (1, 2). This iatrogenic immunosuppression significantly increases susceptibility to opportunistic infections such as cytomegalovirus (CMV) pneumonitis (3, 4). In clinical practice, differentiating drug-induced interstitial lung disease (ILD) from infection remains a major challenge due to overlapping radiologic features (5, 6). This dilemma is worsened by bronchoalveolar lavage next-generation sequencing (BAL-NGS), where findings require rigorous clinicoradiologic correlation to avoid diagnostic misdirection (7). Although CMV pneumonitis has been reported with other tyrosine kinase inhibitor (TKI) classes (e.g., BCR-ABL inhibitors) (8), it is exceptionally rare with osimertinib. A systematic PubMed search for (“osimertinib”) AND (“cytomegalovirus” OR “CMV”) confirmed the absence of prior cases, and its progression to irreversible pulmonary fibrosis has not been described. We present a pivotal case of fulminant CMV pneumonitis that was misdiagnosed as Mycobacterium avium complex (MAC) infection based on BAL-NGS findings in a lymphocytopenic patient, culminating in irreversible fibrosis. This case underscores critical diagnostic pitfalls during TKI therapy and provides a stark clinical example of a lethal “immune-fibrotic axis,” demonstrating the central role of lymphocyte dynamics in driving this fatal trajectory.

## 2 Case presentation

### 2.1 Initial presentation and diagnostic dilemma

**Figure 1:**
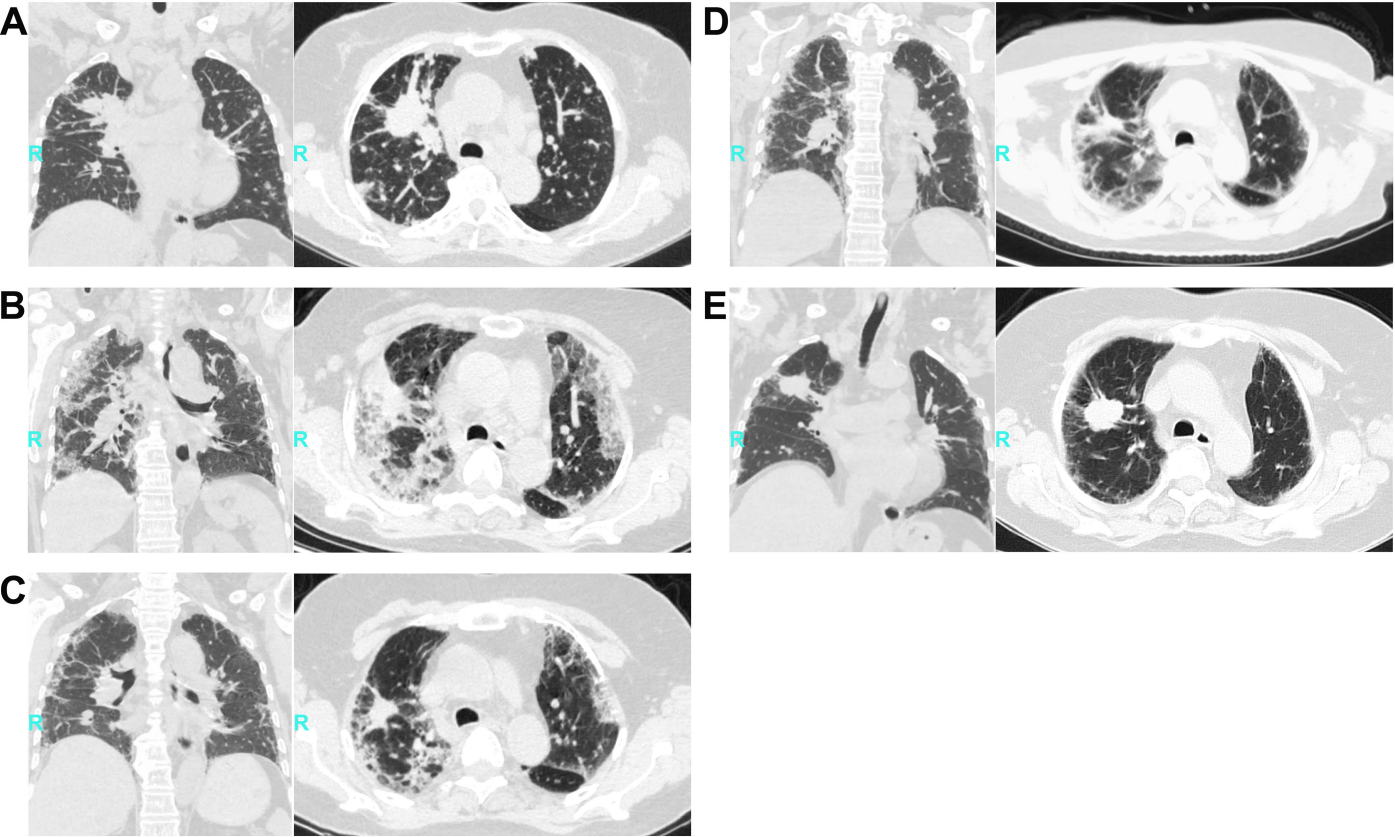
Sequential thoracic CT in EGFR-mutant lung adenocarcinoma with CMV pneumonitis. (All images: lung window [WW 1500, WL -600]). **(A)** Baseline (11 Sep 2023): Spiculated right hilar mass (47×37 mm) with interlobular septal thickening suggests lymphangitic carcinomatosis. **(B)** Acute pneumonitis (28 Dec 2023): Diffuse bilateral ground-glass opacities (GGOs) with septal thickening and reticulation. **(C)** Progressive damage (12 Jan 2024): Increased GGO density with peribronchovascular crazy-paving pattern. **(D)** Fibrosing phase (18 Feb 2024): Subpleural and peribronchovascular consolidations with reticulation. BAL confirmed CMV viremia (20,000 copies/mL). **(E)** Post-inflammatory fibrosis (1 May 2024): Irreversible architectural distortion with traction bronchiectasis. BAL, bronchoalveolar lavage; CMV, cytomegalovirus; DAD, diffuse alveolar damage; EGFR, epidermal growth factor receptor; GGO, ground-glass opacity; TKI, tyrosine kinase inhibitor; WL, window level; WW, window width.

A 75-year-old non-smoking female with stage IV EGFR exon 19 deletion NSCLC lung adenocarcinoma (baseline imaging [11 September 2023]: **Figure 1A**) presented on 28 December 2023 with acute fever, debilitating dyspnea at rest (mMRC grade 4), dry cough, and anorexia 12 days after osimertinib discontinuation (80 mg/day for 1 month). Physical examination revealed tachypnea (28 breaths per minute), tachycardia (110 beats per minute), and coarse crackles on auscultation bilaterally. Thoracic Computed Tomography (CT) revealed new diffuse bilateral ground-glass opacities (GGOs) with smooth interlobular septal thickening (**Figure 1B**), accompanied by profound lymphocytopenia (absolute lymphocyte count [ALC] 0.48 × 10^9^/L [baseline 1.87 × 10^9^/L]) and elevated C-reactive protein (47.81 mg/L). Despite a clinical triad suggestive of infection, the initial diagnosis favored drug-induced ILD, delaying virologic assessment.

### 2.2 Diagnostic misdirection and management missteps

**Figure 2:**
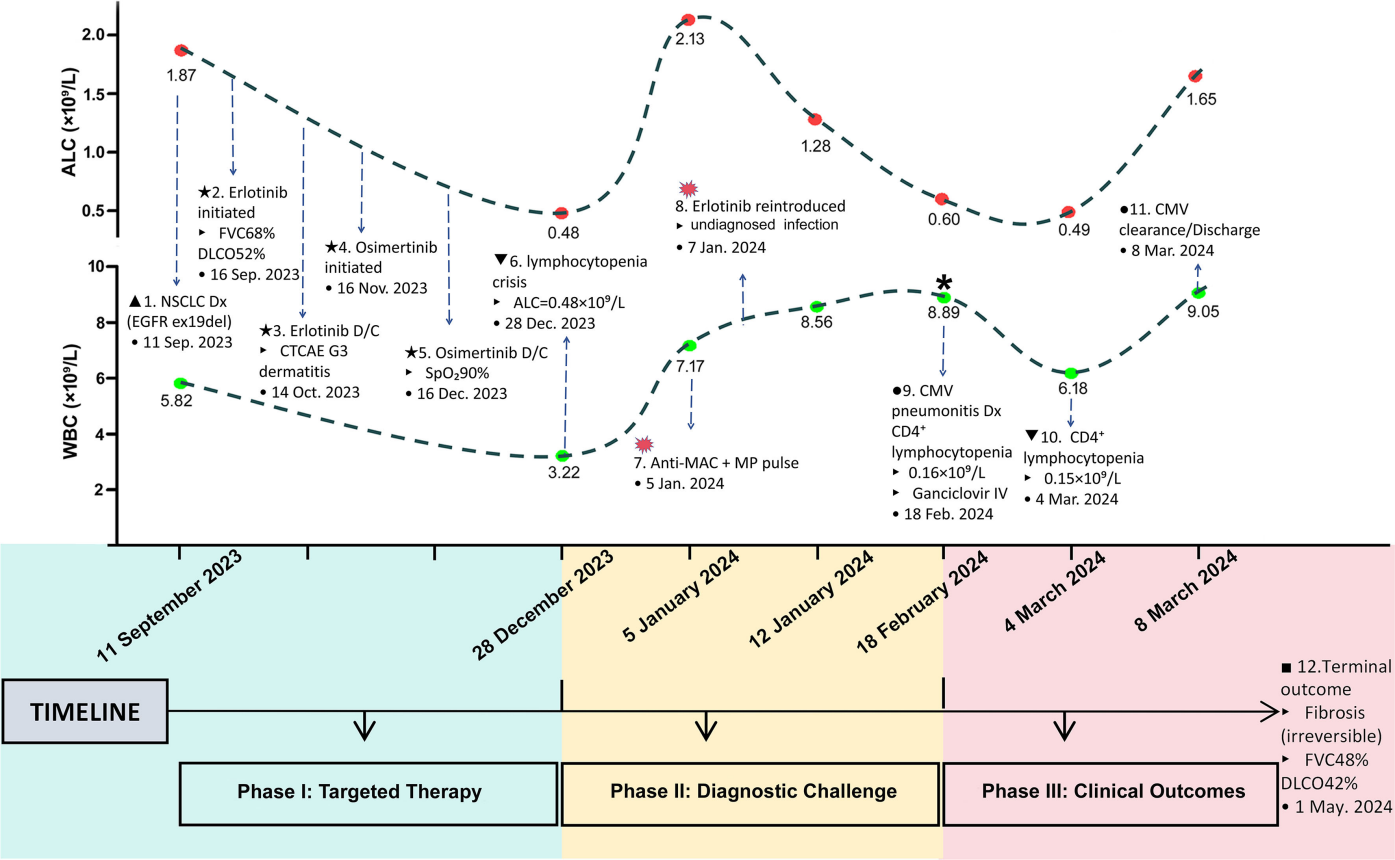
Dynamics of white blood cell, absolute lymphocyte count, and CMV viremia. Serial trends in peripheral WBC and ALC (×10^9^/L). Asterisk (*) indicates BAL-confirmed high-grade CMV viremia (20,000 copies/mL) during a period of profound CD4^+^ lymphocytopenia. Annotated with key clinical events. ALC, absolute lymphocyte count; BAL, bronchoalveolar lavage; CMV, cytomegalovirus; DLCO, diffusing capacity of the lung for carbon monoxide; FVC, forced vital capacity; WBC, white blood cell count.

Although BAL-NGS identified MAC DNA (12,853 copies/mL; 5 January 2024), the BAL cellular profile (neutrophilia 32%) and the absence of cavitation on imaging were inconsistent with typical MAC infection. Subsequent management preceded viral exclusion: viral PCR was not urgently performed despite severe lymphocytopenia (ALC <0.5×10^9^/L), and erlotinib was rechallenged (7 January 2024) before infection exclusion, inducing rapid lymphocyte depletion (ALC decline 2.13→1.28×10^9^/L; 39.9% reduction; see **Figure 2**). Follow-up CT (12 January 2024) showed progressive crazy-paving patterns and increased GGO density, indicating advancing alveolar damage (**Figure 1C**). This was interpreted as stabilization, reinforcing the MAC diagnosis and leading to discharge (16 January 2024) on antimycobacterials.

### 2.3 Clinical deterioration and definitive diagnosis

Acute re-admission occurred on 18 February 2024 (10 days post-erlotinib cessation) with fever, dry cough, hypotension (96/52 mmHg), and hypoxemia (SpO_2_ 88% on room air) requiring urgent oxygen therapy due to incapacitating dyspnea. CT showed peri-bronchovascular consolidations with early reticulation and cord-like shadows (**Figure 1D**). BAL-PCR confirmed high-level CMV viremia (20,000 copies/mL) and profound CD4^+^ lymphocytopenia (0.16×10^9^/L), following a non-diagnostic lung biopsy that had revealed only non-specific interstitial inflammation. This severe immunosuppression likely resulted from the synergistic interplay of erlotinib-induced lymphotoxicity, uncontrolled CMV dissemination, and prolonged corticosteroid exposure (**Figure 2**). Serologic tests for viral hepatitis, EBV, HIV, Leishmania, Cryptococcus, and bunyaviruses were negative. Full-dose ganciclovir (5 mg/kg IV q12h) initiated on 21 February 2024 achieved virologic clearance (<500 copies/mL) by day 7 and complete symptom resolution by day 21.

### 2.4 Treatment response and irreversible sequelae

Despite discharge on 8 March 2024, follow-up CT (1 May 2024) confirmed irreversible fibrotic remodeling with traction bronchiectasis and reticulation (**Figure 1E**), resulting in permanent respiratory impairment (FVC 48%, DLCO 42%) and functional disability, requiring long-term supplemental oxygen (2–4 L/min) for minimal activities of daily living. The stability of these fibrotic changes on a subsequent CT (12 March 2025) confirmed their irreversible nature. A comprehensive chronology is summarized in **Table 1**.

**Table 1:** Clinical timeline of EGFR-mutant NSCLC with treatment complications.

Date	Phase	Event description	Key clinical details
11 Sep 2023	Phase I: Targeted Therapy	Diagnosis confirmed	EGFR exon 19 deletion NSCLC with lymphangitic carcinomatosis
16 Sep 2023		First-line erlotinib initiated	150 mg daily; Baseline pulmonary function: FVC 68%, DLCO 52%
14 Oct 2023		Erlotinib discontinued	Grade 3 exfoliative dermatitis (CTCAE criteria)
16 Nov 2023		Second-line osimertinib initiated	80 mg daily (post-erlotinib intolerance)
7 Dec 2023		Ongoing osimertinib with new symptoms	Anorexia, nausea, dyspnea (mMRC grade 2)
16 Dec 2023		Osimertinib discontinued	Progressive respiratory symptoms with hypoxemia (SpO_2_ 90%)
28 Dec 2023	Phase II: Diagnostic Challenge	Empiric antibiotics initiated	Empiric antibiotics initiated for presumed drug-induced ILD; Nadir lymphocytopenia (ALC = 0.48×10^9^/L)
5 Jan 2024		Anti-MAC therapy + corticosteroids	Despite neutrophilic BAL (32%) and NGS MAC+; Methylprednisolone 1.5mg/kg/day (05–12 Jan); Partial radiographic improvement; CMV investigation deferred
7 Jan 2024		Erlotinib re-initiated	During undiagnosed respiratory infection
12 Jan 2024		Continued erlotinib/antimycobacterials; steroid taper	Methylprednisolone reduced to 0.5mg/kg/day by 25 Jan; Radiographic viral dissemination pattern
8 Feb 2024		Erlotinib discontinued	Recrudescent respiratory symptoms: dyspnea (mMRC grade 4), oxygen dependency
18 Feb 2024	Phase III: Clinical Outcomes	CMV pneumonitis confirmed, BAL PCR: 20,000 copies/mL	Profound CD4^+^ lymphocytopenia (0.16×10^9^/L); IV ganciclovir initiated(21 February 2024); Methylprednisolone 1.0 mg/kg/day
4 Mar 2024		Immunophenotyping performed	Persistent CD4^+^ lymphocytopenia (0.15×10^9^/L); B-cell predominance (31.46%)
8 Mar 2024		Discharge	CMV clearance (viremia <500 copies/mL); Residual dyspnea (mMRC grade 2)
1 May 2024		Palliative care initiation	Home oxygen therapy (2–4 L/min); Irreversible pulmonary fibrosis (FVC 48%, DLCO 42%)

ALC, Absolute Lymphocyte Count; BAL, Bronchoalveolar Lavage; CMV, Cytomegalovirus; CTCAE, Common Terminology Criteria for Adverse Events; DLCO, Diffusing Capacity of the Lung for Carbon Monoxide; EGFR, Epidermal Growth Factor Receptor; FVC, Forced Vital Capacity; MAC, Mycobacterium avium Complex; mMRC, modified Medical Research Council Dyspnea Scale; NGS, Next-Generation Sequencing; NSCLC, Non-Small Cell Lung Cancer; PCR, Polymerase Chain Reaction; SpO_2_, Peripheral Oxygen Saturation; TKI, Tyrosine Kinase Inhibitor.

## 3 Discussion

### 3.1 Diagnostic pitfalls: overreliance on BAL-NGS and delayed viral testing

This case elucidates a critical immune-fibrotic axis wherein TKI-induced lymphocytopenia drives fulminant viral pneumonitis and irreversible pulmonary fibrosis. The interplay between targeted therapy, immunosuppression, and fibrotic remodeling highlights the role of immune homeostasis in lung pathogenesis (9). Retrospectively, the entire clinical course represented a unified trajectory of progressive CMV pneumonitis—initially attributed to drug toxicity then interpreted as MAC infection—rather than discrete events (5, 6). This delay permitted unimpeded viral propagation in an immunocompromised host, which ultimately led to fibrosis.

### 3.2 The keystone mechanism: CD4^+^ T-cell depletion as the central mediator of the immune-fibrotic axis

Osimertinib is clinically associated with a high incidence of severe lymphocytopenia (1, 10), which has been significantly correlated with poorer survival outcomes in patients with EGFR-mutant NSCLC (10, 11). A similar trend of hematologic toxicity is also observed with erlotinib (12). These agents directly induce lymphocyte apoptosis by inhibiting EGFR-dependent survival signals (13–15), which in our patient resulted in profound CD4^+^ T-cell depletion (nadir 0.16×10^9^/L). This created an immunodeficiency state severe enough to be compared to advanced HIV (CD4^+^ <0.2×10^9^/L) (3, 16), crippling antiviral surveillance and permitting uncontrolled CMV replication (3, 4). Beyond infection risk, this lymphocytopenia fundamentally disrupted immune-mediated fibrotic regulation (17). The critical loss of CD4^+^ T cells, particularly regulatory T cells and IFN-γ-producing effectors, removed essential anti-fibrotic brakes. IFN-γ is a potent inhibitor of fibroblast proliferation and collagen synthesis (18, 19); its deficiency created a permissive environment for unchecked fibrogenesis.

### 3.3 Convergent profibrotic pathways: a hypothetical framework

While the present case lacks direct measurements of TGF-β or other profibrotic mediators, the postulated pathways discussed below represent a plausible and literature-supported hypothesis for the observed irreversible fibrotic outcome. Fibrosis progression resulted from the convergence of viral, immunological, and hypoxic pathways within the immunosuppressed microenvironment. CMV infection directly amplified fibrotic signaling by upregulating TGF-β, a master regulator of fibroblast-to-myofibroblast differentiation and ECM deposition (20–23). Concurrently, viral immunopathology and tissue injury promoted M2 macrophage polarization (24) and potentially neutrophil extracellular trap (NET) release (25, 26), generating a cascade of pro-fibrotic mediators including PDGF and CTGF. Persistent alveolar injury led to chronic hypoxia, stabilizing HIF-1α, which synergizes with TGF-β to enhance myofibroblast activity and suppress apoptosis (27, 28). This multifactorial process, unleashed in the context of failed immune regulation, ultimately drove irreversible fibrotic remodeling (29). A schematic summarizing this proposed ‘immune-fibrotic axis’ is provided in **Supplementary Figure S1**.

### 3.4 Clinical implications and proposed management strategies

This case compels a re-evaluation of management strategies for TKI-related pulmonary complications toward an immune-aware approach, particularly since existing guidelines offer no specific directives for this clinical scenario (5, 30). Profound lymphocytopenia (ALC <0.5×10^9^/L) should be recognized not as an incidental laboratory abnormality but as a state of significant immunodeficiency (2, 3, 31, 32). We propose an integrated strategy (**Table 2**) including: (1) urgent CMV and PJP PCR testing in symptomatic, lymphopenic patients before attributing lung injury to drug toxicity (2, 31, 33, 34); (2) avoiding TKI rechallenge during active infection or persistent severe lymphocytopenia (ALC <0.5×10^9^/L)—a critical measure whose omission led to life-threatening CMV pneumonitis here, aligning with NCCN guidelines (5, 30); (3) rigorous clinicoradiologic correlation for interpreting BAL-NGS findings to avoid diagnostic errors (5, 7, 30, 35); and (4) serial immune monitoring (ALC and CD4^+^ counts) for risk stratification and early intervention (1, 2, 4, 10). Future studies should investigate whether prophylactic or pre-emptive antiviral strategies can mitigate lung injury in this high-risk population. The patient’s perspective is reflected in her functional decline (mMRC dyspnea scale) and the profound impact on her quality of life, evidenced by permanent supplemental oxygen requirement for minimal daily activities and subsequent transition to palliative care.

**Table 2:** Summary of clinical scenario, pitfall, and recommended action.

Clinical scenario	Pitfall & consequence	Recommended action	Evidence
Suspected TKI-ILD with severe lymphocytopenia (ALC = 0.48×10^9^/L; 28 Dec 2023)	Omission of CMV/PJP PCR despite ALC<0.5×10^9^/L; Consequence: 52-day delayed CMV diagnosis; uncontrolled viral replication (20,000 copies/mL).	Recommend immediate CMV/PJP PCR testing in any lymphocytopenic (ALC<0.5×10^9^/L) patient before attributing injury to drug toxicity.	(2, 3, 31, 32)
BAL-NGS detection of MAC DNA (12,853 copies/mL; 5 Jan 2024)	Prioritizing NGS results without clinical/radiographic validation (no cavitation; BAL neutrophils 32%); Consequence: delayed treatment, and unnecessary antimycobacterial therapy.	Recommend microbiologic confirmation (culture/serology) and radiographic correlation before initiating treatment based solely on NGS findings.	(5, 7, 30, 35)
TKI rechallenge decision with persistent lymphocytopenia (7 Jan 2024)	Erlotinib reintroduction during: Profound immunosuppression (ALC 0.48×10^9^/L); Undiagnosed CMV pneumonitis; Consequence: Rapid lymphocyte depletion (ALC↓39.9%); Viral dissemination → irreversible pulmonary fibrosis.	Advise against TKI rechallenge during active infection or persistent severe lymphocytopenia (ALC<0.5×10^9^/L).	(5, 30)
Underlying immunological risk state (Throughout treatment course)	Underlying Risk (Throughout): Profound, progressive TKI-induced CD4+ lymphocytopenia (nadir 0.16×10^9^/L) was the central mediator of the entire fatal trajectory. Rationale: Proactive monitoring could enable pre-emptive intervention before complications arise.	Suggest serial immune monitoring (ALC and CD4+ counts) for risk stratification in patients on high-risk TKIs.	(1, 2, 4, 10)

ALC, absolute lymphocyte count; BAL, bronchoalveolar lavage; CMV, cytomegalovirus; DLCO, diffusing capacity of the lung for carbon monoxide; ILD, interstitial lung disease; MAC, Mycobacterium avium complex; NGS, next-generation sequencing; PJP, *Pneumocystis jirovecii* pneumonia; SpO_2_, peripheral oxygen saturation; TKI, tyrosine kinase inhibitor.

In conclusion, despite the limitations of a single-case retrospective design and non-diagnostic histopathology, this case illustrates how targeted therapy disrupts lymphocyte dynamics, permitting opportunistic infections and initiating a self-perpetuating cycle of inflammatory fibrosis. Understanding this immune-fibrotic axis is key to developing improved preventive and therapeutic strategies.

## Data Availability

The original contributions presented in the study are included in the article/**Supplementary Material**. Further inquiries can be directed to the corresponding author.

## Supplementary material

Supplementary Figure 1 Proposed “Immune-Fibrotic Axis”. The hypothesized cascade from TKI-induced CD4^+^ T-cell depletion leads to uncontrolled cytomegalovirus (CMV) pneumonitis, driving profibrotic signaling and irreversible pulmonary fibrosis. ECM, Extracellular Matrix; NET, Neutrophil Extracellular Trap.
