# Supplementary figures and images for: Lymphocyte dynamics as the central mediator in osimertinib-induced CD4+ T-cell depletion, fulminant cytomegalovirus pneumonitis, and progressive pulmonary fibrosis: a case report
Source: Front Immunol. 2025 Dec 17;16:1702074. doi: 10.3389/fimmu.2025.1702074 (PMC12753373; doi:10.3389/fimmu.2025.1702074)

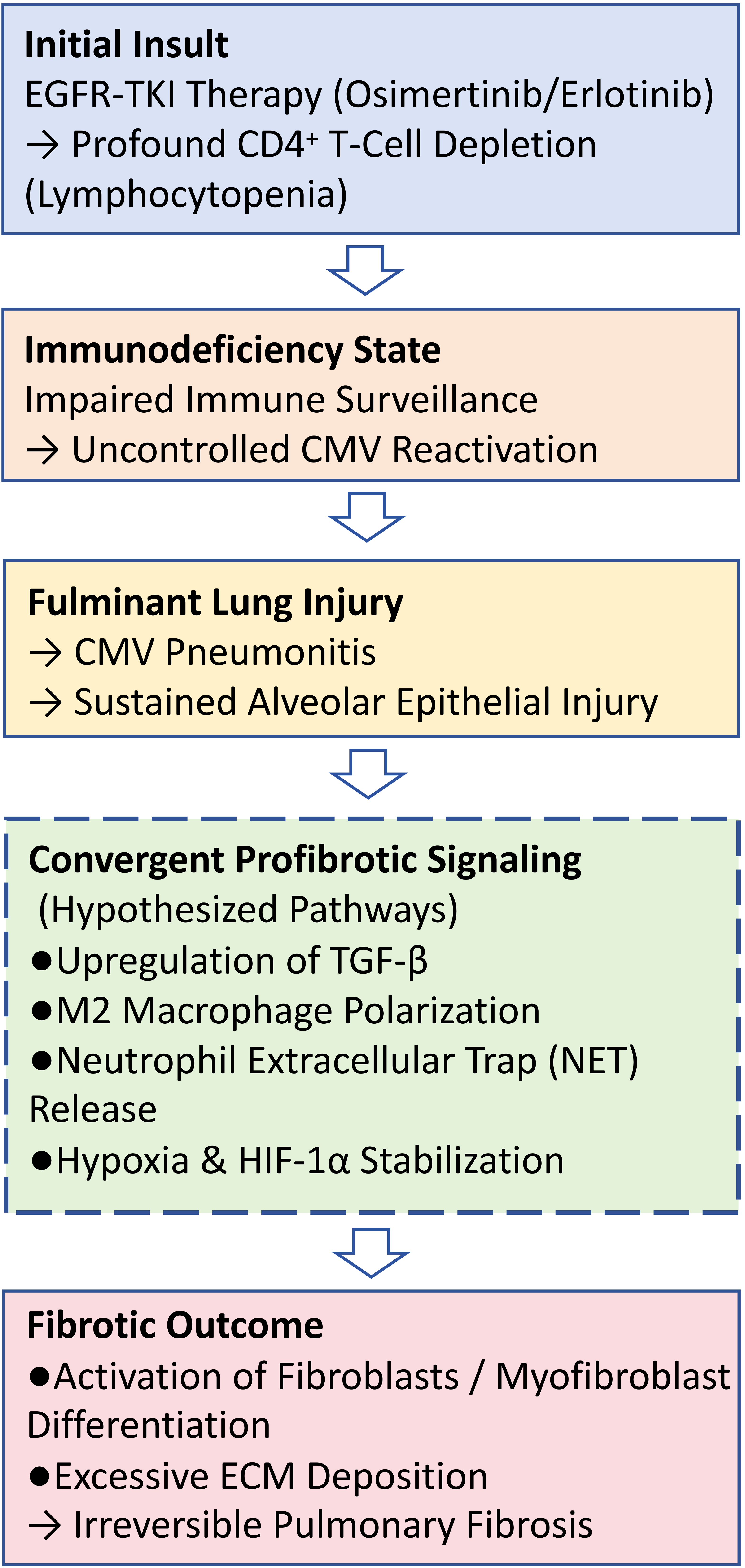

Supplement: Supplementary Figure 1 — Proposed “Immune-Fibrotic Axis”. The hypothesized cascade from TKI-induced CD4+ T-cell depletion leads to uncontrolled cytomegalovirus (CMV) pneumonitis, driving profibrotic signaling and irreversible pulmonary fibrosis. ECM, Extracellular Matrix; NET, Neutrophil Extracellular Trap. [file Image1.tif]
